# The complete mitochondrial genome of *Scatophagus argus* (Perciformes, Scatophagidae) from Beibu Bay and phylogenetic analysis

**DOI:** 10.1080/23802359.2021.1959445

**Published:** 2021-08-02

**Authors:** Chuanyan Pan, Chongmin Gao, Tao Chen, Chunling Yang, Digang Zeng, Pengfei Feng, Weiming Jiang, Xiuli Chen, Min Peng

**Affiliations:** a Guangxi Key Laboratory of Aquatic Genetic Breeding and Healthy Aquaculture, Nanning, China; b Guangxi Academy of Fishery Sciences, Nanning, China; c Guangxi Agricultural Vocational College, Nanning, China

**Keywords:** Mitochondrial genome, *Scatophagus argus*, Scatophagidae

## Abstract

The spotted scat (*Scatophagus argus*, Linnaeus, 1766) is a subtropical fish that is widely distributed in the coastal waters of Indo-Pacific. Here, we report the complete mitochondrial genome of *S. argus*. The mitogenome is 16,783 base pairs (56.0% A + T content) in length and consists of 13 protein-coding genes, 22 tRNA genes, 2 rRNA genes and a 1007 bp D-loop region. Phylogenetic analysis showed that the relationship between *S. argus* and *Selenotoca multifasciata* was close.

The spotted scat (*Scatophagus argus*) is distributed in the coastal waters of the Indo-Pacific, South and South East Asia, China, and Russia (Kharin and Milovankin 2008; Chen et al. 2015). It has become a favorite food fish due to its high nutritional value and delicious taste (Gupta 2016). *S. argus* uses a wide range of food sources. It has strong adaptability to environment and strong disease resistance. It can grow and develop in brackish water and fresh water, so it is easy to breed, and the breeding cost is low (Gupta 2016). Therefore, it has become an economically important aquaculture species in China’s coastal waters (Lan et al. 2005). The complete mitochondrial genomes of *S. argus* from Yangjiang, Guangdong Province and Jiangsu Province have been reported. However, little genetic data is available for *S. argus* from Beibu Bay. The complete mitochondrial genome is an excellent molecular marker for studying phylogenetic relationships and species identification (Zhong et al. 2020). The purpose of this study was to determine the complete mitochondrial genome of *S. argus*.

The research materials were collected in Weizhou Island (21.027201 N, 109.133285E), Beihai city, Guangxi province, China. The whole body specimens (#BH201905310003) were deposited at Guangxi Key Laboratory of Aquatic Genetic Breeding and Healthy Aquaculture, Nanning, China. The total genomic DNA from the muscle of an individual was extracted via the phenolchloroform extraction method (Kumar and Mugunthan 2018). DNA libraries were constructed using the TruSeq NanoTM kit and were sequenced on a HiSeq platform. Mitogenome assembly was performed using the MITObim software. Gene annotation was performed using the MITOS software (http://mitos2.bioinf.uni-leipzig.de/). The phylogenetic tree was constructed using MEGA X (https://www.megasoftware.net/) with maximum-likelihood method, and the bootstrap replicates parameter was set to 1000.

The complete mitogenome of *S. argus* collected from Beibu bay was found to be 16,783 bp in length (GenBank accession number: MN909969) with the base composition of A (28.3%), T (27.7%), C (28.2%) and G (15.8%). The mitogenome length of *S. argus* collected from Beibu bay was shorter than *S. argus* collected from Jiangsu province(16,778 bp). The percentage of G + C was 44.0% and the percentage of A + T was 56.0%, which were the same as those of *S. argus* collected from Yangjiang (Guangdong province) (Liu et al. 2014) and lower than those of *Selenotoca multifasciata* (45.5% of G + C and 54.5% of A + T) (Liu et al. 2016) and *S. argus* collected from Jiangsu province (44.6% of G + C and 55.4% of A + T) (Chen et al. 2015). The mitogenome contains 13 protein-coding genes, 22 tRNA genes, 2 rRNA genes (a 12S rRNA and a 16S rRNA) and a control region. The control region was 1007 bp and was located between a tRNA-Pro gene and a tRNA-Phe gene.

In the ML phylogenetic tree, *S. argus* from Beibu bay was first clustered with *S. argus* collected from Yangjiang, Guangdong province, China. Then, *it* was clustered with *Selenotoca multifasciata*, which were classified into Scatophagidae family. *Siganus guttatus*, *Siganus sutor*, *Siganus canaliculatus*, and *Siganus fuscescens* formed another clade, which were classified into Siganidae family. *Ephippus orbis*, *Platax orbicularis* and *Platax teira* formed another clade, which were classified into Ephippidae family (Figure 1). The results showed that Scatophagidae has the closer relationship with Ephippidae thanSiganidae. This study will enrich the genome data of Scatophagidae, and will be useful for resources conservation and management of *S. argus*.

**Figure 1.**
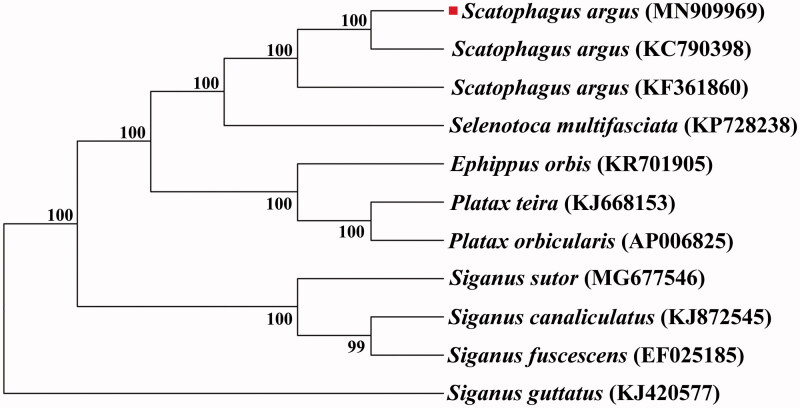
A phylogenetic tree was constructed based on the comparison of mitochondrial genome sequences of *S. argus* and other species of Perciformes. All the sequences were downloaded from NCBI GenBank. *Note.* The red square on the figure 1 represents the sample for this study.

## Data Availability

The genome sequence data that support the findings of this study are openly available in GenBank of NCBI at (https://www.ncbi.nlm.nih.gov/) under the accession no. MN909969. The associated BioProject, SRA, and Bio-Sample numbers are PRJNA688809, SRR13336106, and SAMN17189441, respectively."

## References

[CIT0001] Chen JH, Li YL, He MX, Yan BL, Meng XP. 2015. Complete mitochondrial genome of the spotted scat *Scatophagus argus* (Teleostei, Scatophagidae). Mitochondrial DNA. 26(2):325–326. 25835066 10.3109/19401736.2013.830295

[CIT0002] Gupta S. 2016. An overview on morphology, biology, and culture of spotted scat *Scatophagus argus* (Linnaeus 1766). Rev Fish Sci Aquacult. 24(2):203–212.

[CIT0003] Kharin VE, Milovankin PG. 2008. A new finding of rare species *Scatophagus argus* (Scatophagidae) in Russian waters. J Ichthyol. 48(9):822–824.

[CIT0004] Kumar M, Mugunthan M. 2018. Evaluation of three DNA extraction methods from fungal cultures. Med J Armed Forces India. 74(4):333–336. 30449918 10.1016/j.mjafi.2017.07.009 PMC6224647

[CIT0005] Lan GB, Yan B, Liao SM, Luo Y, Xie RC. 2005. Biology of Spotted Scat *Scatophagus argus*: a review. Fish Sci. 24(7):39–41.

[CIT0006] Liu ZH, Mu XJ, Li H, Gui L, Zeng WG, Zhang JB. 2016. Complete mitochondrial genome of the striped scat *Selenotoca multifasciata* (Perciformes: Scatophagidae). Mitochondrial DNA A DNA Mapp Seq Anal. 27(4):2691–2692. 27158788 10.3109/19401736.2015.1046125

[CIT0007] Liu ZZ, Zhang TR, Su LW, Zhang JB, Yang JQ. 2014. Complete mitochondrial genome of the spotted scat *Scatophagus argus (Perciformes: Scatophagidae)*). Mitochondrial DNA. 25(6):422–423. 23834083 10.3109/19401736.2013.809439

[CIT0008] Zhong SP, Huang LH, Liu YH, Huang GQ, Chen XL. 2020. The complete mitochondrial genome of *Phascolosoma similis* (Sipuncula, Phascolosomatidae) from Beibu Bay. Mitochondrial DNA B. 5(2):1263–1264.

